# Machine learning and bioinformatics-based insights into the potential targets of saponins in *Paris polyphylla* smith against non-small cell lung cancer

**DOI:** 10.3389/fgene.2022.1005896

**Published:** 2022-10-28

**Authors:** Yue Wang, Xulong Huang, Bin Xian, Huajuan Jiang, Tao Zhou, Siyu Chen, Feiyan Wen, Jin Pei

**Affiliations:** State Key Laboratory of Southwestern Chinese Medicine Resources, School of Pharmacy, Chengdu University of Traditional Chinese Medicine, Chengdu, China

**Keywords:** bioinformatics, key genes, Paris polyphylla smith, non-small cell lung cancer, machine learning

## Abstract

**Background:** Lung cancer has the highest mortality rate among cancers worldwide, and non-small cell lung cancer (NSCLC) is the major lethal factor. Saponins in Paris polyphylla smith exhibit antitumor activity against non-small cell lung cancer, but their targets are not fully understood.

**Methods:** In this study, we used differential gene analysis, lasso regression analysis and support vector machine recursive feature elimination (SVM-RFE) to screen potential key genes for NSCLC by using relevant datasets from the GEO database. The accuracy of the signature genes was verified by using ROC curves and gene expression values. Screening of potential active ingredients for the treatment of NSCLC by molecular docking of the reported active ingredients of saponins in *Paris polyphylla* Smith with the screened signature genes. The activity of the screened components and their effects on key genes expression were further validated by CCK-8, flow cytometry (apoptosis and cycling) and qPCR.

**Results:** 204 differential genes and two key genes (RHEBL1, RNPC3) stood out in the bioinformatics analysis. Overall survival (OS), First-progression survival (FP) and post-progression survival (PPS) analysis revealed that low expression of RHEBL1 and high expression of RNPC3 indicated good prognosis. In addition, Polyphyllin VI(PPVI) and Protodioscin (Prot) effectively inhibited the proliferation of non-small cell lung cancer cell line with IC50 of 4.46 μM ± 0.69 μM and 8.09 μM ± 0.67μM, respectively. The number of apoptotic cells increased significantly with increasing concentrations of PPVI and Prot. Prot induces G1/G0 phase cell cycle arrest and PPVI induces G2/M phase cell cycle arrest. After PPVI and Prot acted on this cell line for 48 h, the expression of RHEBL1 and RNPC3 was found to be consistent with the results of bioinformatics analysis.

**Conclusion:** This study identified two potential key genes (RHEBL1 and RNPC3) in NSCLC. Additionally, PPVI and Prot may act on RHEBL1 and RNPC3 to affect NSCLC. Our findings provide a reference for clinical treatment of NSCLC.

## Introduction

Lung cancer is the leading cause of cancer deaths (18.4% of all cancer deaths), with more than 2.1 million lung cancer cases (725,352 in women and 1,368,524 in men) and more than 1.76 million deaths in 2018 (Thai et al., 2021). It has the third highest incidence rate among female cancer patients and the first highest incidence rate among male cancer patients worldwide (Bray et al., 2018). Approximately 85% of lung cancers are non-small cell lung cancer (NSCLC), which mainly includes lung adenocarcinoma (LUAD), lung squamous carcinoma (LUSC), and large cell carcinoma (LCC) (Herbst et al., 2018), but most NSCLC patients are diagnosed with advanced stages of the disease due to the limitations of current screening technologies (Ettinger et al., 2017). Despite the advances in targeted drug therapy and surgery for NSCLC, the desired survival rates are still not achieved (Hirsch et al., 2017). Therefore, it is valuable to combine the large amount of disease-related bioinformatics data available to uncover novel predictors for prognosis and potential therapeutic targets for NSCLC research and treatment (Mangogna et al., 2019).

In recent years, the study of disease mechanisms and the exploration of relevant disease-characterizing genes as well as anticancer drug targets with the help of metabolomics (Li, 2015; Hurgobin et al., 2018; Hong et al., 2020) have made good progress, especially the prediction of potential targets (Zhu et al., 2012) and relevant biomarkers (Zheng et al., 2021), which has greatly advanced the field. Compared with time-consuming and expensive traditional acting target studies, bioinformatics analysis combined with machine learning can screen potential acting targets more rapidly and accurately, and it provides exploratory predictions at a lower cost to inform subsequent conducted biological experiments and clinical applications (Sepulveda, 2020; Talukder et al., 2021).

In some studies of non-small cell lung cancer (Ni and Sun, 2019; Wang et al., 2020; Li et al., 2021), TTC21 was found to be highly expressed in lung adenocarcinoma by public database analysis, achieving a good prognosis (Wang et al., 2020). Promising therapeutic targets for LUAD were revealed, including genes such as CDK1, CDH1, CDKN3, CDKN2A, CD34, IL6, FOS, MMP9, VWF, EDN1, BIRC5, UBE2C, AURKA, CCBN2, and EGR1 (Ni and Sun, 2019). PIWIL4, IFIT1B, 8IGF2BP1 TLR8, PABPC1, ZC3H12C, PECAM1, ENG, and GAPDH were identified as core genes for the construction of prognostic models (Li et al., 2020; Wang et al., 2021). Although several biomarkers or targets for NSCLC have been identified in recent years. However, the sensitivity and specificity of these biomarkers or therapeutic targets lack further validation due to sample heterogeneity and many confounding factors. Therefore, in-depth experimental validation analysis of novel prognostic predictors and therapeutic targets is needed in the future.

*Paris polyphylla* smith is used by folk medical practitioners in China and India to treat a variety of lung diseases, including consumption, pneumonia, and lung cancer (Liu et al., 2019; Yan et al., 2021). The anticancer activity of the saponins in *Paris polyphylla* smith has received increasing attention in recent years. Polyphyllin VI (PPVI)has been reported to induce Caspase-1-mediated scorching through induction of the ROS/NF-κB/NLRP3/GSDMD signaling axis in NSCLC (Teng et al., 2020). It has also been found that PPVI induces apoptosis and autophagy in NSCLC through the ROS-triggered mTOR signaling pathway (Teng et al., 2019). In addition, it could induce the accumulation of ROS in cells and down-regulated the Bcl-2 expression, up-regulated the Bax expression and induced the activity of cleaved caspase-3 in cells (You et al., 2021). Moreover, PPVI can be used in a variety of tumor cells such as osteosarcoma, hepatocellular carcinoma, and glioma by regulating ROS/JNK(Yuan et al., 2019), Fas death pathway and mitochondria-dependent pathway (Liu et al., 2018), and JNK/P38 (Liu et al., 2020). Protodioscin also has inhibitory effects on non-small cell lung cancer cell lines (Hu and Yao, 2002), but the anti-non-small cell lung cancer research of protodioscin basis is extremely weak.

In this study, we searched for differential genes through gene expression data published in GEO, and explored possible pathways using Disease Ontology (DO), Gene Ontology (GO) and Kyoto Encyclopedia of Genes and Genomes (KEGG) enrichment analysis after normalization of differential genes; further screened and validated potential signature genes RHEBL1 and RNPC3 in non-small cell lung cancer. The correlation between the expression of signature genes and the prognosis of patients with NSCLC was evaluated using KM-plotter and GEIPA database, and it was found that high expression of RNPC3 and low expression of RHEBL1 significantly prolonged the survival cycle of patients, and the key genes were analyzed for immune infiltration. By molecularly docking the bioactive saponins of *paris polyphylla* smith with the corresponding proteins of RHEBL1 and RNPC3, Molecular docking screening of compounds Polyphyllin VI and protodioscin with affinity ≤ −7 kcal/mol. And the toxicity, apoptosis and cycle of Polyphyllin VI and protodioscin on A549 cells were verified by CCK-8, flow cytometry. We also verified the effects of Polyphyllin VI and protodioscin on toxicity, apoptosis and cycle of A549 cells by CCK-8, Flow Cytometric Analysis. Also using qPCR, we found that RNPC3 gene expression was significantly upregulated and RHEBL1 gene expression was significantly downregulated in A549 cells after 48 h of Polyphyllin VI and protodioscin action, which was consistent with the results of bioinformatics analysis. This study provides evidence for a new potential clinical strategy for non-small cell lung cancer.

## Material and methods

### Gene expression omnibus and differential gene analysis

We searched Gene Expression Omnibus (http://www.ncbi.nlm.nih.gov/geo/, accessed on 3 January 2022) for eligible datasets meeting the criteria and downloaded the platform files and series matrix files about GSE33532, GSE151103, and GSE44077. Find the correspondence between probe names and gene names based on the information in the platform file, and use Perl scripts to convert the probe matrix into gene expression matrix. The combined data files were subjected to differential analysis by limma software package, and the differential judgment criterion was | log FC(Fold Change)| > 1 with False Discovery Rate< 0.01 (Deng et al., 2021; Ebrahimi Sadrabadi et al., 2021).

GSE151103 samples are from 126 patients with stage IA-IV non-small cell lung cancer, including tissue samples from 188 patients with non-small cell lung cancer (133 LUAD samples and 55 LUSC samples), and 172 matched normal tissue samples. GSE33532 included lung cancer tissue samples (80 cases) and matched distant normal lung tissue (20 cases) from 20 patients with stage I-II non-small cell lung cancer (16 men and 4 women, including 11 cases of lung adenocarcinoma, 4 cases of lung squamous cell carcinoma and 5 cases of mixed type). GSE44077 samples were taken from tissue samples from 20 patients with stage I-III a NSCLC (*n* = 9 women and 11 men) (including 14 lung adenocarcinoma, 5 lung squamous cell carcinoma and 1 unspecified non-small cell lung cancer). Multiple cytologically controlled normal airways and unaffected normal lung tissues were different from the tumor (*n* = 194 samples). GSE33532 and GSE151103 were used as train sets, GSE44077 as validation set.

### Gene ontology term and kyoto encyclopedia of genes and genomes and disease ontology pathway enrichment analysis

GO, KEGG, and DO analyses were used to explore the enrichment of differential genes. Through Gene Ontology, we divided the function of genes into three parts: cellular component (CC), molecular function (MF) and biological process (BP). KEGG enrichment analysis was performed to observe in which pathways the differential genes were enriched, and the corresponding bar and bubble plots were completed using the barplot and ggplot2 software packages. DO analysis of differential genes was performed by clusterProfiler, org. Hs.eg.db, enrichplot, GSEABase, and DOSE packages to explore in which diseases differential genes were significantly enriched. GO, KEGG and DO enrichment analyses were performed with *p* < 0.05 as cutoff values (Wang et al., 2022).

### Screening of feature genes

Least absolute regression and selection operator (Lasso regression) can adjust the variables and complexity when fitting the generalized linear model, and can effectively avoid overfitting. It is suitable for multivariate discrete, continuous or multivariate dependent variables. Lasso regression and support vector machine recursive feature elimination (SVM-RFE) were used to screen the characteristic genes of non-small cell lung cancer, and then R-packet Venn was used to obtain the two overlapping genes to further screen the diagnostic genes of non-small cell lung cancer. R-Packet limma and ggpubr were used to verify whether the feature genes were different in the validation set (GSE44077).

### ROC curve and test ROC curve

Through receiver operating characteristic curve (ROC curve), we can observe the accuracy of the genes screened by machine learning as the diagnostic genes of non-small cell lung cancer. We used R-packet pROC to draw ROC curves (Huang et al., 2022).

### Estimation of immune cell infiltration in related patterns

The immunocyte infiltration was analyzed by cibersort software package (https://cibersort.stanford.edu/), and the results were screened under the condition of *p* < 0.05 (Tang et al., 2022). The results were used to analyze the correlation between immune cells. At the same time, the difference of immune cells and the correlation between target genes and immune cells were analyzed. It is executed by R package e1071, corrlot, barlot, violot, reshape2, ggpubr, and ggExtra.

### Kaplan-meier plotter and gene expression profiling interactive analysis

We used Kaplan-Meier (KM) plotter (http://kmplot.com) to explore the potential prognostic significance of feature genes in non-small cell lung cancer. Survival analysis included Overall survival (OS), first-progression survival (FP) and post-progression survival (PPS). In addition, we used the “Correlation Analysis” module of the GEPIA database (http://gepia.cancer-pku.cn/index.html) to determine the correlation between RHEBL1 and RNPC3 in LUAD and LUSC based on the TCGA-LUAD and TCGA-LUSC data in this database. In this study, we determined the correlation between RHEBL1 and RNPC3 in LUAD and LUSC, respectively, using Spearman’s correlation coefficient (Zhou et al., 2021).

### Molecular docking

The relevant proteins were downloaded from the PDB Protein Structure Database (www.pdbus.org). RHEBL1 X-ray diffraction (PDB ID: 3OES), RNPC3 solution NMR (PDB ID: 5OBN). Discovery Studio 2016 (Version16.1.0) processed the relevant proteins and the relevant saponin-like components using autodock to dock the relevant small molecule to the protein batch three times (Trott and Olson, 2010), and the docking score was Affinity, with smaller values indicating stronger binding. The following rule of thumb (rule of thumb) is usually used. Affinity ≤ −7 kcal/mol is more strongly bound (Huang et al., 2022).

### Chemicals and materials

Protodioscin, Polyphyllin VI were provided by Push bio-technology (Chengdu, China), Cisplatin was provided by RHAWN (Shanghai, China). Dulbecco’s Modified Eagle’s Medium (DMEM) was obtained from Hyclone (Logan, UT, United States). Fetal bovine serum (FBS) was obtained from Every Green (Zhejiang, China), Penicillin Streptomycin Solution was obtained from Hyclone (Logan, UT, United States). TransZol Up Plus RNA Kit was purchased from TransGen Biotech (Beijing, China), PrimeScript RT Reagent Kit was purchased from Takara Bio (Shiga Prefecture, Japan).

### Cell culture and cell viability assays

A549 was purchased from the ATCC (Manassas, VA, United States). Cells were cultured at 37°C and 5% CO_2_. DMEM contained penicillin (100 U/ml), streptomycin (100 g/1), and FBS (10%). Cell Counting Kit-8 (CCK-8) assay was used to determine cell viability. Cells were seeded in 96-well plates (100 μl/well). The density of A549 was 5 × 10^3^ cells/well. After treating the cells with different concentrations of Polyphyllin VI (14 μM, 7 μM, 3.5 μM, 1.75 μM, 0.875 μM, and 0.4375 μM), protodioscin (75 μM, 50 μM, 25 μM, 12.5 μM, and 6.25 μM)and cisplatin (2 μM) for 48 h, respectively, the control group was set. Subsequently, the cell viability was measured by CCK-8 assay according to the manufacturer’s instructions.

### Apoptosis analysis and cell-cycle analysis

Apoptosis was detected by the Annexin V-FITC/PI Apoptosis Kit. To collect A549 cells treated with different protodioscin (75 μM, 50 μM, and 25 μM), Polyphyllin VI(14 μM, 7 μM, and 3.5 μM)and Cisplatin (2 μM)for 48 h. A total of 500 μl Binding buffer, 5 μl Annexin V-FITC and 10 μl PI was added in sequence in the dark, after mixing and standing for 15 min, the cell fluorescence was detected by flow cytometry (BD Biosciences, CA, United States).

Multi Sciences Cell Cycle Staining Kit was used to determine Cell cycle. A549 cells were treated with protodioscin (75 μM, 50 μM, and 25 μM), Polyphyllin VI (14 μM, 7 μM, and 3.5 μM) and Cisplatin (2 μM) for 48 h. 1 ml DNA Staining solution and 10 μl Permeabilization solution was added for staining. Incubate for 30 min in the dark, and then immediately detect the cell cycle by flow cytometry (BD Biosciences, CA, United States).

### RNA isolation and real-time PCR

Total RNA of A549 was extracted using the TransZol Up Plus RNA Kit according to the instructions, reverse transcription was performed using the PrimeScript TM RT reagent Kit with gDNA Eraser (Perfect Real Time), and the Bio-RAD CFX96TM Real- Time System for quantitative PCR, RHEBL1 primer (forward, 5′-TACC GCT​GTG​TAG​GGA​AGA​CA-3′, reverse, 5′-CCA​CTG​TAG​GAT​CGT​AGC​CTT-3′), RNPC3 primer (forward, 5′-GTG​CGG​GTC​CTG​TCA​GAT​AAG-3′, reverse, 5′-TGA​ACT​CGA​TCT​TGC​TCT​TTT​GC-3′), and GAPDH (forward, 5′-GGAGCGAGA TCCCTCCCCAAAAT-3′, reverse 5′-GGC​TGT​TGT​CAT​ACT​TCT​CAT​GG-3′). Real-time PCR was performed in triplicate using samples derived from three independent experiments.

### Statistical analysis

All statistical analyses were performed with R software version 4.1.3 (https://www.r-project.org/) and SPSS 25.0 software (IBM, Armonk, NY, United States) for pre-processing and analysis of public data, including sample data merging, ID transformation and duplicate removal. In addition, Pearson correlations were used to assess correlations between genes and genes, and genes and associated immune infiltrating cells. Differences between the two groups were assessed using *t*-test (for normally distributed data) or Mann-Whitney test (for non-normal distribution) and *p* < 0.05 was considered statistically significant. Graphs were constructed using R software and online drawing tools as well as Graphpad (https://www.graphpad.com/).

## Results

### Analysis of differential genes

**FIGURE 1:**
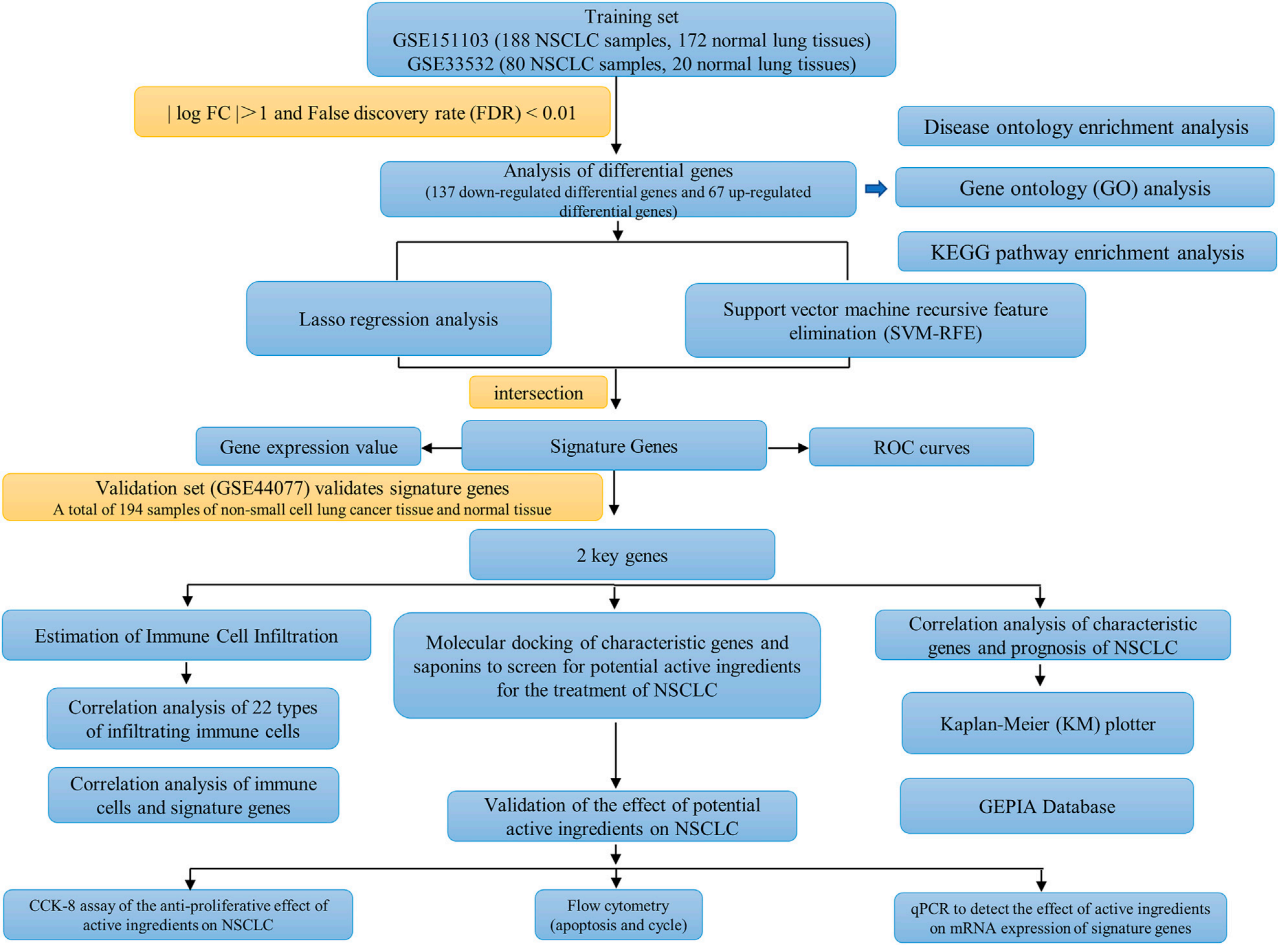
The workflow of this study.

**FIGURE 2:**
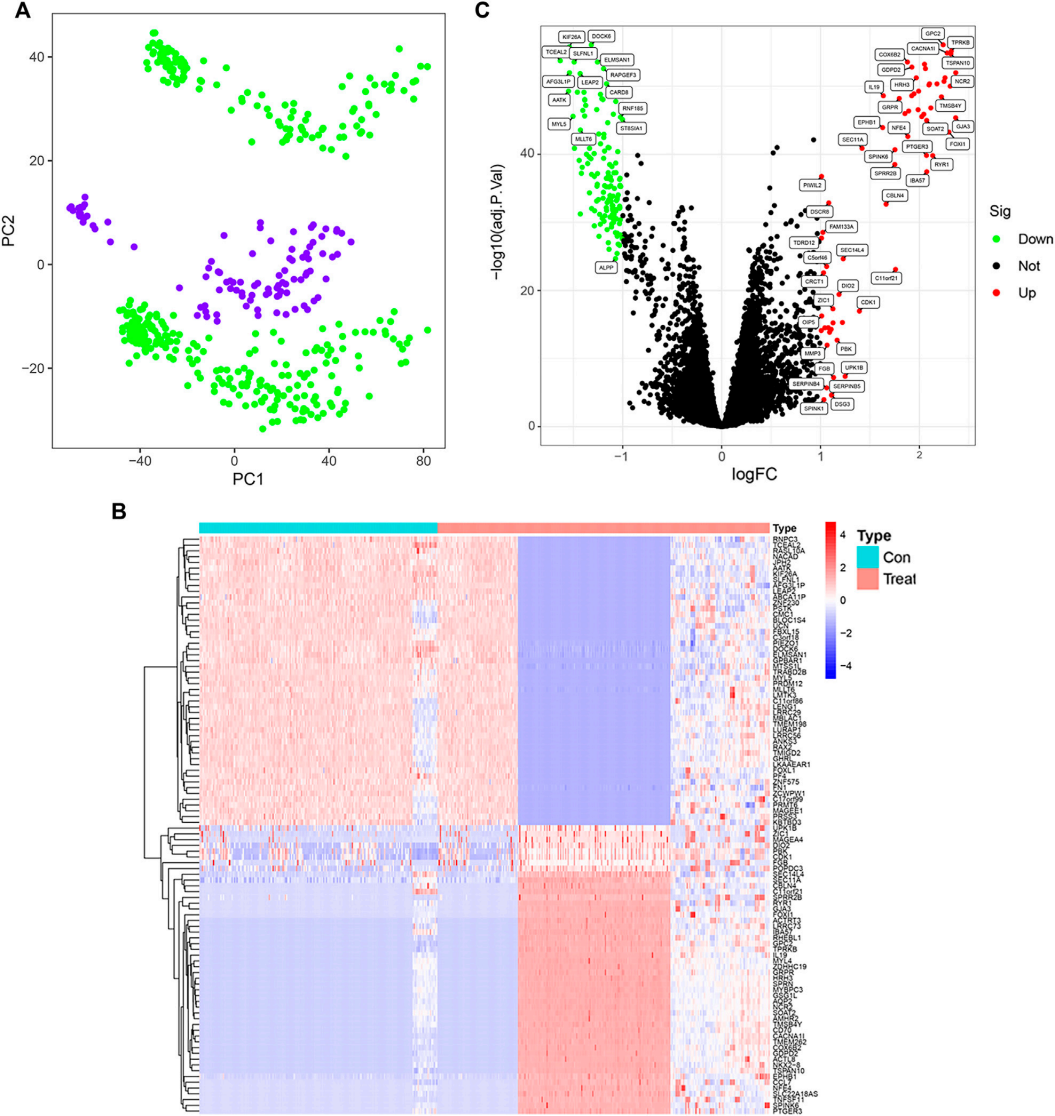
Screening of differential genes in non-small cell lung cancer control and treatment groups. **(A)** GSE151103, GSE33532 data normalization (PC1 on the *x*-axis represents the first principal component, and PC2 on the *y*-axis represents the second principal component). **(B)** Heatmap of differentially expressed genes (Red indicates up-regulated expression of the gene and blue indicates down-regulated expression of the gene). **(C)** Differential gene expression visualization (red circles indicate genes with up-regulated expression, green circles indicate genes with down-regulated expression, and black circles indicate genes with no change).

The workflow of this study is shown in Figure 1. A total of 204 differential genes for non-small cell lung cancer were screened by differential gene analysis, 137 differential genes were down-regulated and 67 differential genes were up-regulated. The data were normalized by limma and sva packages (Figure 2A). The 50 most significantly up-regulated differential genes and the 50 most significantly down-regulated differential genes were plotted as a heat map (Figure 2B). and then three packages, dplyr, ggplot2, and ggrepel, were cited, and the genes with significant differences were labeled with names to visualize the differential genes (Figure 2C).

### Enrichment analysis of differential genes in patients with non-small cell lung cancer

**FIGURE 3:**
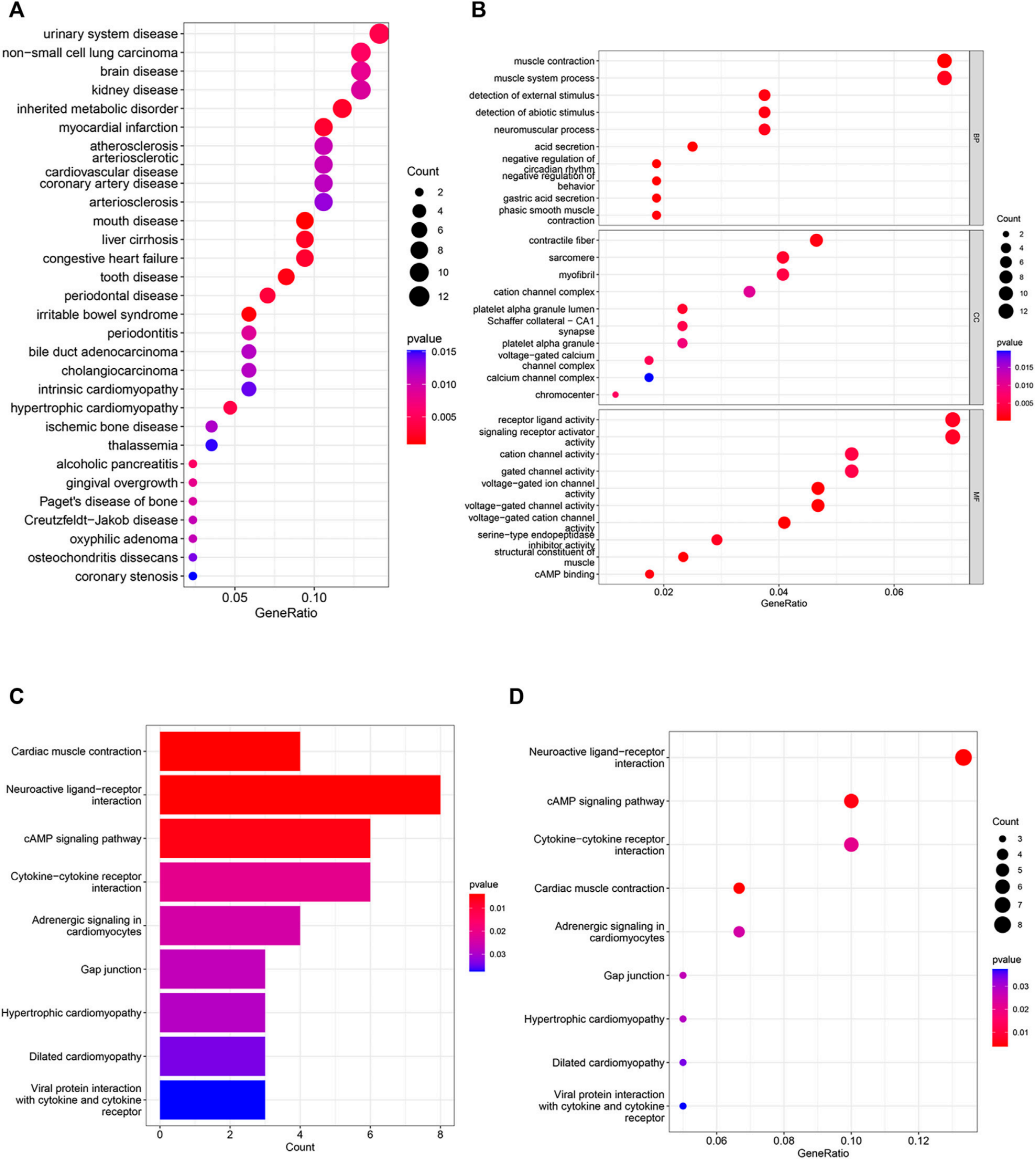
DO, GO and KEGG analyses in differential genes. **(A)** 30 categories of diseases associated with differential genes based on DO analysis. **(B)** GO analysis shows the signaling pathways enriched in BP, MF and CC in NSCLC. (CC: cellular component, MF: molecular function, BP: biological process, the color represents the significance of the data, the redder the color means the more significant the differential genes are enriched in this pathway). **(C–D)** Analysis of KEGG-based enriched signaling pathway.

DO analysis revealed (Figure 3A) that urological diseases, non-small cell lung cancer, and brain diseases topped the list of terms. The results of GO analysis (Figure 3B; Supplementary Figure S1) showed that in BP enrichment analysis, the screened differential genes were significantly enriched in muscle contraction, detection of abiotic stimulus, negative regulation of circadian rhythm; CC enrichment analysis showed significant enrichment in contractile fibers, cation channel complex and voltage-gated calcium channel complex; MF enrichment analysis showed significant enrichment in receptor ligand activity, signaling receptor activator activity and cation channel activity.

The results of KEGG enrichment analysis showed (Figures 3C,D) that NSCLC differential genes were significantly enriched in Neuroactive ligand-receptor interactions, CAMP signaling pathways, Cytokine-cytokine receptor interactions were significantly enriched in these pathways, and these pathways accounted for the top three in the KEGG correspondence. Cytokines control immune-related events, and cytokines play a crucial role in the immune regulation of disease and are involved in plethora of physiological and pathophysiological processes, including cancer development and autoimmunity (Spangler et al., 2015; Scheller et al., 2019). The above pathways strongly suggest that non-small cell lung cancer is closely associated with immune cells.

### Screening for non-small cell lung cancer key genes

**FIGURE 4:**
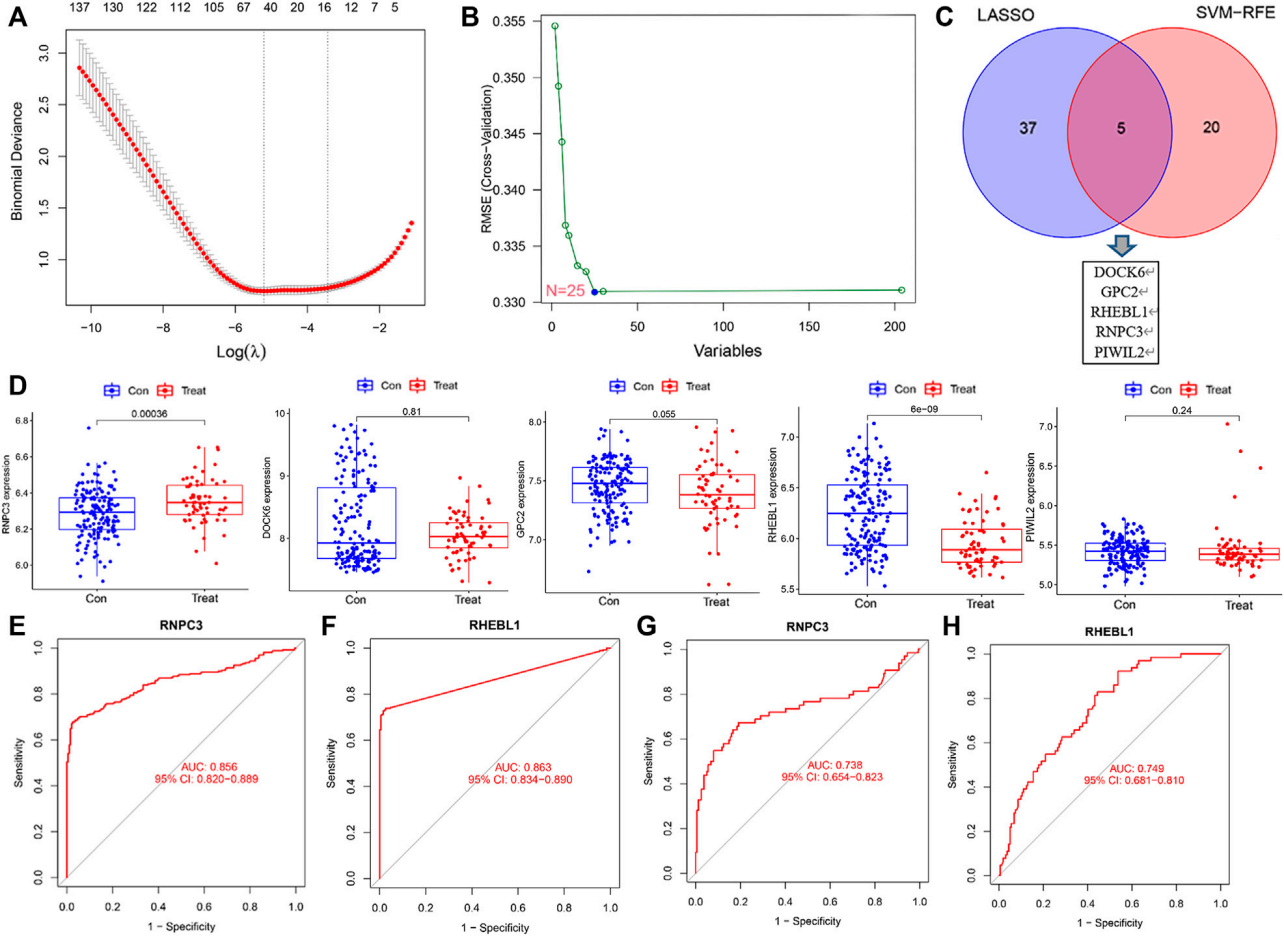
Screening and validation of feature genes. **(A)** LASSO regression for 42 differentially expressed genes (Vertical coordinate: cross-validation error. λ is the adjustment parameter. Red dots indicate partial likelihood deviance, gray lines indicate standard errors (SE), and the two vertical dashed lines on the left and right side indicate the best values of the minimum and 1-SE criteria, respectively). **(B)** SVM-RFE screening for 25 differentially expressed genes. **(C)** Intersection results of LASSO and SVM-RFE algorithms. **(D)** Expression of 5 genes in the control and treatment group (Expression of RNPC3, RHEBL1 was significantly different between the control and treatment group. *p* < 0.001). **(E–F)** Receiver operating characteristic curves for signature genes (the area under the curve for RNPC3 and RHEBL1 was greater than 0.85, indicating the high accuracy of these genes as diagnostic genes for non-small cell lung cancer). **(G–H)** The ROC curve of the validation set (GSE44077)was further introduced to verify the accuracy of RNPC3, RHEBL1 as diagnostic genes for NSCLC.

To identify signature genes associated with clinical features of non-small cell lung cancer patients, a total of 42 non-small cell lung cancer signature genes were screened by Lasso regression analysis using the training sets GSE151103 and GSE33532 (Figure 4A; Supplementary Figure S2). Meanwhile, 25 disease signature genes were screened by support vector machine recursive feature elimination (SVM-RFE) (Figure 4B; Supplementary Figure S3). The intersection of the two was taken to screen five non-small cell lung cancer signature genes from the training sets GSE151103 and GSE33532: DOCK6, GPC2, RHEBL1, RNPC3, and PIWIL2 (Figure 4C). The introduction of the validation set GSE44077 to validate the five genes screened (Figure 4D) revealed that the expression levels of RNPC3 (*p* = 0.00036) in lung cancer tissues of non-small cell lung cancer were significantly higher than those of matched normal controls, and the expression levels of RHEBL1 (*p* = 6e-0.9) were significantly lower than those of matched controls; while DOCK6 (*p* = 0.81), PIWIL2 (0.24) and GPC (0.055)were not significantly different between the disease and normal control groups. P< 0.05 was considered a significant difference.

The above screening revealed that the expression levels of RNPC3 and RHEBL1 differed significantly in treatment and control group. To further verify the accuracy of these differential genes as diagnostic genes for non-small cell lung cancer, ROC curve analysis of the above these genes was applied and found that RNPC3 (AUC: 0.856, 95% CI: 0.820–0.889), and RHEBL1 (AUC: 0.863, 95% CI: 0.834–0.890) all had AUCs greater than 0.8 (Figures 4E,F). The validation set (GSE44077) was further included in the test ROC, and the results showed that the AUC of RNPC3 (AUC: 0.738, 95% CI: 0.654–0.823) and RHEBL1 (AUC: 0.749, 95% CI: 0.681–0.810) was still greater than 0.7 (Figures 4G,H). It has been found that RHEBL1 is involved in sphingosylphosphorylcholine-induced events in A549 lung cancer cells *via* binding to AKT1 leading to activation of it (Kim et al., 2017). In addition, anti-RNPC3 antibody can be used as a marker for cancer scleroderma including lung cancer (Shah et al., 2017). This suggests a high probability of diagnosing non-small cell lung cancer with RNPC3 and RHEBL1.

### Correlation analysis between expression of key genes and prognosis of patients with non-small cell lung cancer

**FIGURE 5:**
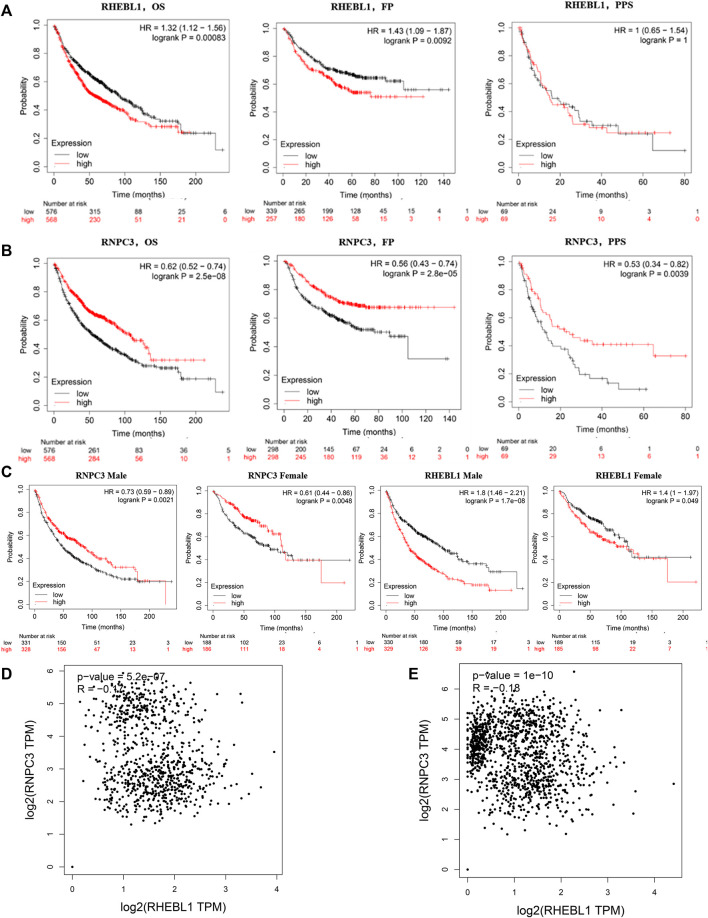
Survival curves based on different expression levels of RHEBL1 and RNPC3 in different subgroups of non-small cell lung cancer patients. **(A,B)** Performance of RHEBL1 and RNPC3 in OS, FP and PPS of non-small cell lung cancer (OS: Overall survival, FP: first-progression survival, PPS: post-progression survival. In OS, FP, high expression of RHEBL1 showed unfavorable prognosis. In addition, high expression of RNPC3 in OS, FP, PPS showed favorable prognosis). **(C)** Effect of RNPC3 and RHEBL1 expression levels in different genders on the prognosis of NSCLC patients. **(D–E)** Correlation scatter plot of RHEBL1, RNPC3 in LUSC and LUAD (RNPC3 and RHEBL1 show negative correlation in LUSC and LUAD).

The above analysis showed that the expression of the feature gene RNPC3 was significantly higher in the treatment group and RHEBL1 was significantly lower in the treatment group compared to the control group. Further analysis of the effect of the expression status of the signature gene on the prognosis of non-small cell patients by Kaplan-Meier (KM) plotter, compared with the high expression group, the low expression group of RHEBL1 in non-small cell lung cancer patients (including LUAD and LUSC) showed significantly favorable in OS, FP. Unfortunately, the high and low RHEBL1 gene in PPS expression did not differ significantly in terms of prognosis (Figure 5A). In contrast, low expression of RNPC3 was significantly associated with poor prognosis in OS, FP and PPS (Figure 5B). Further breakdown by gender revealed that high expression of RNPC3 in female and male related patients (LUAD, LUSC) was significantly associated with a good prognosis in related patients. low expression of RHEBL1 was significantly associated with favorable survival in both male and female NSCLC patients (Figure 5C). Correlation analysis by the GEPIA database revealed a significant negative correlation between RHEBL1 and RNPC3 in LUAD and LUSC, which is consistent with the above results (Figures 5D,E). In conclusion, low expression of RHEBL1 or high expression of RNPC3 was significantly associated with good prognosis in patients with non-small cell lung cancer.

### Immune infiltration associated with patients with non-small cell lung cancer

**FIGURE 6:**
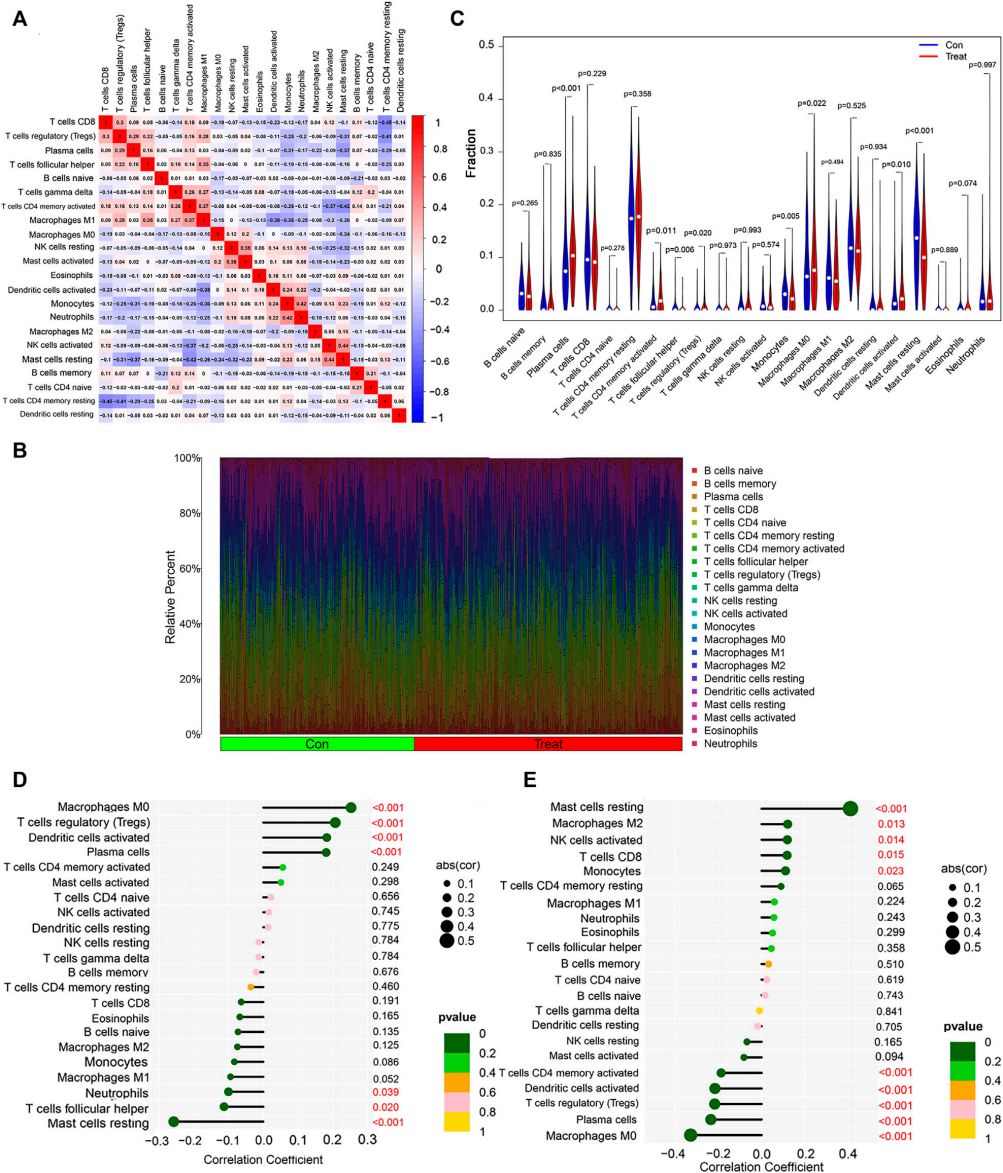
Immune infiltration analysis of non-small cell lung cancer. **(A)** Correlation analysis among 22 immune cell types. **(B)** Visualization of the degree of infiltration of multiple immune cells in non-small cell lung cancer samples. **(C)** Changes in the distribution of 22 immune cell types in non-small cell lung cancer tissues and their controls. **(D)** Relationship between RHEBL1 and the level of immune cell infiltration, where red color is statistically significant, *p* < 0.05. **(E)** Relationship between RNPC3 and the level of immune cell infiltration, where red color is statistically significant, *p* < 0.05.

Increasing results suggest that tumor-infiltrating immune cells are potential predictors for patients with non-small cell lung cancer (Koyama et al., 2016; He et al., 2017; Chiou et al., 2021). KEGG functional enrichment showed that non-small cell lung cancer was enriched in immune-related pathways. Therefore, the correlation between 22 immune cells was further analyzed to explore their relationship and possible interactions. The highest positive correlation was found between NK cells activated and Mast cells resting (Pearson’s correlation = 0.44), followed by the correlation between Monocytes and Neutrophils (Pearson’s correlation = 0.42). In terms of negative correlation, T cells CD8 was significantly negatively correlated with T cells CD4 memory Resting (Pearson’s correlation = −0.45) (Figure 6A).

To explore the immune differences between the non-small cell lung cancer group and matched normal controls, we evaluated the expression of GSE151103, GSE33532 datasets with 22 infiltrating immune cells such as B cells naive, B cells memory, Plasma cells, T cells CD8, T cells CD4 naïve relationship (Figure 6B), and further differential analysis of immune cells, immune infiltration results showed that lung tissues of non-small cell lung cancer patients with Plasma cells (*p* < 0.001), T cells CD4 memory activated (*p* = 0.011), T cells regulatory (Tregs) (*p* = 0.020), Macrophages M0 (*p* = 0.022), Dendritic cells activated (*p* = 0.010) were significantly upregulated, T cells follicular helper (*p* = 0.006), Monocytes (*p* = 0.005), Mast cells resting (*p* < 0.001) were significantly downregulated (Figure 6C); finally, the correlation analysis between RNPC3, RHEBL1 and immune cells was explored (Figures 6D,E; Supplementary Figure S4), and RHEBL1 was significantly downregulated with Macrophages M0, T cells regulatory (Tregs), Dendritic cells activated, and Plasma cells were significantly positively correlated with immune cells such as Neutrophils, T cells follicular helper, and Mast cells resting; RNPC3 was significantly and negatively correlated with Mast cells resting, RNPC3 was significantly and positively correlated with immune cells such as Macrophages M2, NK cells activated, T cells CD8, Monocytes, and with Macrophages M0, Plasma cells, T cells regulatory (Tregs), Dendritic cells activated, T cells CD4 memory activated, and other immune cells. In conclusion, the expression of RHEBL1 and RNPC3 significantly correlated with immune cells infiltrated by tumors in patients with non-small cell lung cancer.

### Results of molecular docking of saponins with key genes

**FIGURE 7:**
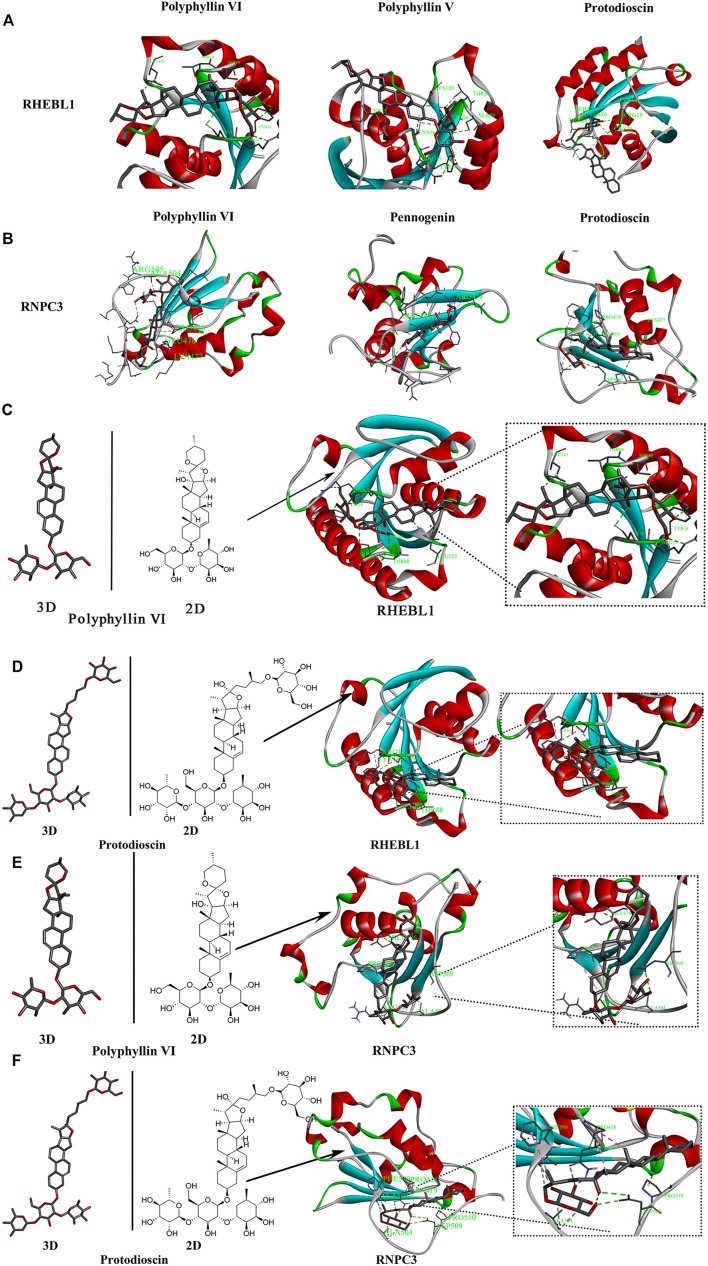
Molecular docking screening of relevant active compounds. **(A,B)** Active compounds with docking scores of −7 kcal/mol to −10 kcal/mol with RHEBL1 and RNPC3 proteins. **(C,D)** Molecular docking of two components (Polyphyllin VI, Protodioscin)and RHEBL1 protein to screen for potential active sites (Polyphyllin VI: LEU123, THR88, SER89, TYR14, ARG15, CYS16. Protodioscin: SER89, THR88, SER86, CYS16, ARG15, TYR14). **(E,F)** Molecular docking of two components and RNPC3 protein to screen for potential active sites (Polyphyllin VI: ARG505, ALA504 PRO418, GLU476, LYS477. Protodioscin: ARG502, ALA501, ASN419, PRO418, ASP509, LYS477).

The saponins with good biological activity in *paris polyphylla* smith were selected for this molecular docking (Supplementary Table S1), and the saponins were docked with RNPC3 (PDB ID: 5OBN) and RHEBL1 (PDB ID: 3oes) using Vina software for three times. The docking results of compounds with docking scores in the range of −7 kcal/mol–−10 kcal/mol with both RNPC3 and RHEBL1 were plotted. The molecular docking results showed that the average scores of RHEBL1 with protodioscin, Polyphyllin VI, and Polyphyllin V docked three times were −8.4, −7.2, and −7.2, respectively (Figure 7A), the average scores of RNPC3 docked three times with protodioscin, Polyphyllin VI, and Pennogenin were −9.3, −7.6, and −7.5 (Figure 7B). From the docking results, it can be seen that Polyphyllin VI: LEU123, THR88, SER89, TYR14, ARG15, and CYS16 are key amino acid residues in the active site of RHEBL1 (Figure 7C). Protodioscin: SER89, THR88, SER86, CYS16, ARG15, and TYR14 are the key amino acid residues of the active site of RHEBL1 (Figure 7D). Polyphyllin VI: ARG505, ALA504 PRO418, GLU476, and LYS477 are key amino acid residues in RNPC3 (Figure 7E). Protodioscin: ARG502, ALA501, ASN419, PRO418, ASP509, and LYS477 are the key amino acid residues of the RNPC3 active site (Figure 7F).

### Inhibition rate of polyphyllin VI and protodioscin and promotion of apoptosis in A549 cells

**FIGURE 8:**
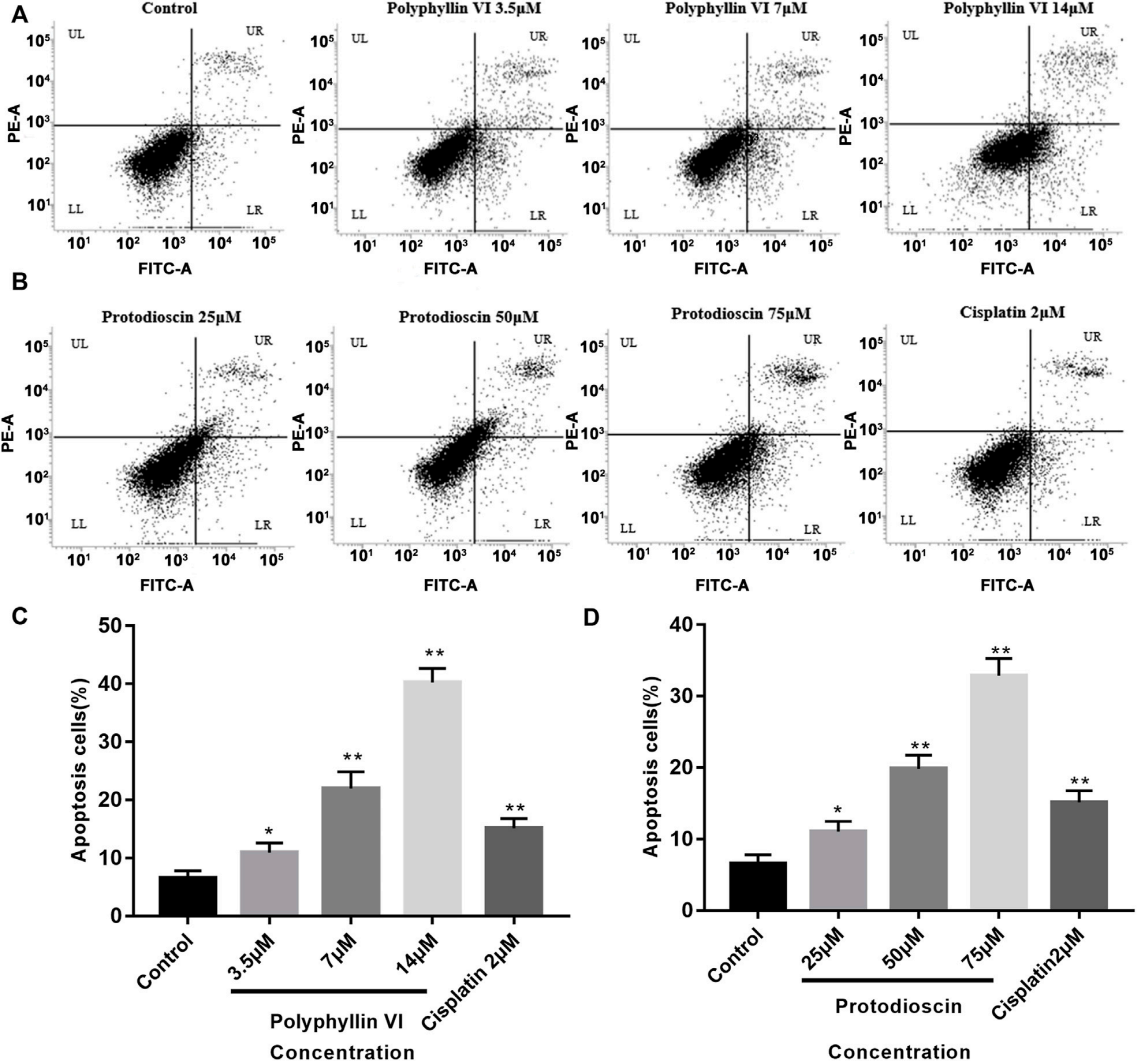
Polyphyllin VI and Protodioscin induce apoptosis of A549 cells. **(A,B)** Apoptosis was detected by annexin V/PI staining. UL represents necrotic cells, LL represents normal cells, UR represents late apoptotic cells, and LR represents early apoptotic cells. **(C,D)** The data show that treatment of A549 cells with Polyphyllin VI, Protodioscin and Cisplatin for 48 h significantly induced apoptosis (*p < 0.05, **p < 0.01, compared with the control group).

We evaluated the inhibitory effects of Polyphyllin VI and Protodioscin on A549 cell line using CCK-8 assay and set cisplatin as positive control group and tested 48 h after administration. As shown in Table 1, Polyphyllin VI and Protodioscin effectively inhibited the proliferation of non-small cell lung cancer cell line A549 with IC50 of 4.46 μM ± 0.69 μM and 8.09 μM ± 0.67 μM, respectively. Further, Annexin V-FITC/PI staining and flow cytometry were used to detect apoptosis. The results showed that the number of normal cells (LL quadrant) gradually decreased and the number of apoptotic cells (UR quadrant, LR quadrant) gradually increased with increasing concentrations of Polyphyllin VI (3.5 μM, 7 μM, and 14 μM). 14 μM Polyphyllin VI and 2 μM Cisplatin treated groups increased from 6.57% ± 1.24% to 40.26% ± 2.44% and 15.14% ± 1.66% of cells, respectively (**p < 0.01, Figures 8A,C). The number of apoptotic cells increased significantly with increasing concentrations of Protodioscin (25 μM, 50 μM, 75 μM), where the percentage of cells in the 75 μM Protodioscin and positive group cisplatin (2 μM) increased from 6.57% ± 1.24% to 32.89% ± 2.39% and 15.14% ± 1.66% (**p < 0.01, Figures 8B,D). These data showed that Polyphyllin VI and Protodioscin induced apoptosis of A549 cells.

**TABLE 1:** Effects of Polyphyllin VI and Protodioscin treatment on IC50 (μM) of A549 cells.

Substance	Cell name	Cell type	IC50(μM)
Polyphyllin VI	A549	Human non-small cell lung cancer cells	4.46 ± 0.69
Protodioscin	A549	Human non-small cell lung cancer cells	8.09 ± 0.67

### Protodioscin induces G1/G0 phase cell cycle arrest and Polyphyllin VI induces G2/M phase cell cycle arrest in A549 cells

**FIGURE 9:**
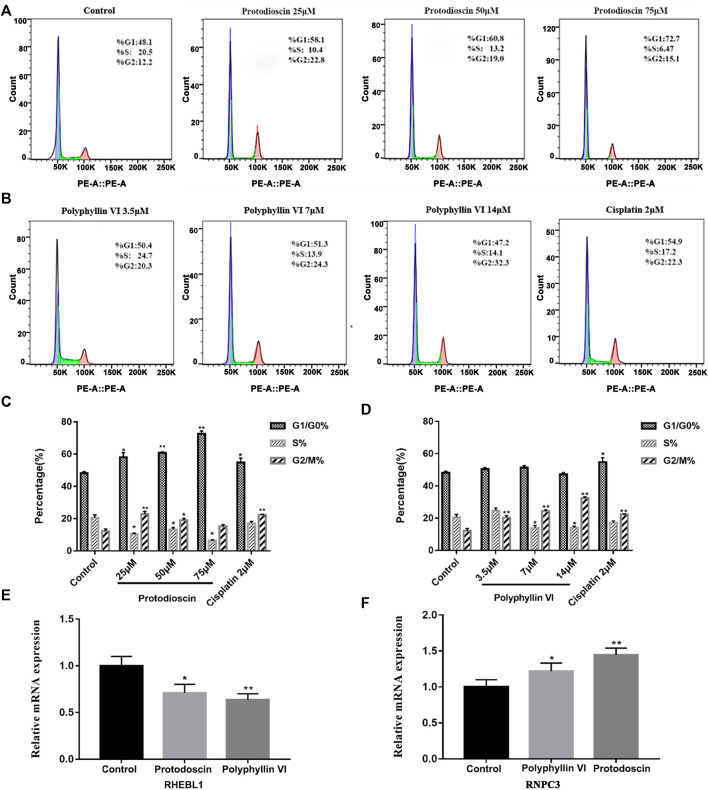
Polyphyllin VI and protodioscin induce cell cycle arrest in A549 cells and the effect on mRNA expression of RHEBL1 and RNPC3 in this cell line. **(A,B)** The cell cycle distribution map of A549 cells exposure to Polyphyllin VI, Protodioscin and Cisplatin for 48 h was detected by flow cytometry. **(C,D)** The data show that Protodioscin (concentration-dependent manner) and Cisplatin significantly increase the ratio of cells in G1/G0 phase. However, Polyphyllin VI (concentration-dependent manner) significantly increase the ratio of cells in G2/M phase. *p < 0.05, **p < 0.01 compared with the control group. Each column represents the mean ± SD of three independent experiments. **(E)** RHEBL1 expression was significantly down-regulated in A549 cells treated with Protodioscin (50 μM) and Polyphyllin VI (3.5 μM) for 48 h; **(F)** RNPC3 expression was significantly upregulated in A549 cells after 48 h treatment with Protodioscin (50 μM) and Polyphyllin VI (3.5 μM). (Results were analyzed using 2^−ΔΔCT^ Method, *p < 0.05, **p < 0.01, compared with the control group. Each column represents the mean ± SD of three independent experiments).

The cell cycle was detected by BD flow cytometry after treating A549 cells for 48 h with Polyphyllin VI, protodioscin and cisplatin, and the results showed that 25 μM, 50 μM, and 75 μM protodioscin stalled the A549 cell cycle in G1/G0 phase, with the 75 μM protodioscin group increasing the ratio from 48. 13% ± 1.04% to 72.69% ± 1.71% (*p < 0.05, **p < 0.01), and the 2 μM cisplatin group also significantly induced A549 cell cycle arrest in the G1/G0 phase (Figures 9A,C).

Interestingly, in A549 cells, the percentage of Polyphyllin VI in G2/M phase exhibited a dose-dependent increase accompanied by increasing Polyphyllin VI concentrations (3.5 μM, 7 μM, and 14 μM). 14μM Polyphyllin VI, 2 μM cisplatin-treated A549 cells exhibited a dose-dependent increase, with a significant increase in the percentage of cells in G2/M phase compared to control (*p < 0.05, **p < 0.01, Figures 9B,D). The above results suggest that protodioscin dose-dependently induced A549 cell cycle arrest in the G1/G0 phase, whereas Polyphyllin VI blocked the A549 cell cycle in the G2/M phase.

### Effects of polyphyllin VI and protodioscin on mRNA of RHEBL1 and RNPC3-related genes in A549 cells

We identified the characteristic genes RHEBL1 and RNPC3 in non-small cell lung cancer by characteristic differential gene analysis, and immuno-infiltration analysis revealed that simultaneous low expression of RHEBL1 or high expression of RNPC3 significantly prolonged the survival time of related patients. Therefore, we further validated our findings by quantitative PCR, which showed that after 48 h of Polyphyllin VI and Protodioscin action on A549 cells, Protodioscin and Polyphyllin VI significantly promoted the low expression of RHEBL1 compared to the control group (*p < 0.05, * *p < 0.01, Figure 9E), while for RNPC3, the relative mRNA expression of Protodioscin and Polyphyllin VI was significantly higher after acting on A549 (*p < 0.05, **p < 0.01, Figure 9F). This is consistent with the results analyzed in GEO, KM-plotter and GEPIA databases. Overall, Protodioscin and Polyphyllin VI significantly promoted either low expression of RHEBL1 or high expression of RNPC3, causing patients with non-small cell lung cancer to exhibit a good prognosis.

## Discussion

Among malignancies, lung cancer has the highest incidence and is the leading cause of cancer death in men and women worldwide. Despite advances in early diagnosis of lung cancer and in targeted therapies, lung cancer is mostly diagnosed at advanced stages and patient survival is low. Therefore, it is important to identify effective prognostic signature genes for lung cancer. In this study, we screened 204 differential genes for non-small cell lung cancer by bioinformatics analysis of the non-small cell lung cancer-related datasets GSE33532, GSE151103, and GSE44077 in the GEO public database (Figure 2). Differential genes were found to be associated with lung diseases, including non-small cell lung cancer, by DO analysis. By GO enrichment analysis and KEGG enrichment analysis, these differential genes were found to be closely associated with immune cells. By GO enrichment analysis and KEGG enrichment analysis, the screened differential genes were found to be closely associated with immune cells (Figure 3). The differential genes were further screened by Lasso analysis and support vector machine recursive feature elimination (SVM-RFE) analysis of the training sets GSE33532 and GSE151103, and verified by ROC curve analysis, which revealed that the expression levels of two genes, RNPC3 and RHEBL1, were significantly different in non-small cell lung cancer tissues and controls (Figure 4). Further testing revealed that low expression of RHEBL1 and high expression of RNPC3 in non-small cell lung cancer significantly improved the prognosis and prolonged life expectancy of cancer patients (Figure 5). KEGG enrichment analysis implied that these differential genes were associated with immune cells, so 22 immune infiltrating cells were introduced to explore their relationship with RHEBL1 and RNPC3, and the results showed that both were significantly associated with immune cells infiltrating tumors in patients with non-small cell lung cancer (Figure 6). The results of bioinformatics analysis, molecular docking, flow cytometry and quantitative PCR suggest that RHEBL1 and RNPC3 may be potential targets for Polyphyllin VI and Protodioscin in the treatment of non-small cell lung cancer.

Polyphyllin VI and Protodioscin with affinity ≤ −7 kcal/mol were selected by molecular docking of the two characterized genes screened with the active ingredients in *paris polyphylla* smith and found by CCK-8 assay that Polyphyllin VI and Protodioscin both inhibited the A549 cell line (Polyphyllin VI: IC50 = 4.46 ± 0.69; Protodioscin: IC50 = 8.09 ± 0.67). Polyphyllin VI have been found to be effective in the treatment of non-small cell lung cancer through the p53 pathway and death receptor pathway (Lin et al., 2015), the ROS/NF-κB/NLRP3/GSDMD signaling axis, and the mTOR signaling pathway (Teng et al., 2019). We detected apoptosis in A549 cells by flow cytometry and found a significant increase in the proportion of apoptotic cells accompanied by increasing concentrations of both compounds; in flow cycle assays, we found that Protodioscin dose-dependently induced A549 cell cycle arrest in G1/G0 phase, while Polyphyllin VI blocked A549 cell cycle arrest in G2/M phase. Further analysis revealed that both substances inhibited the proliferation of the A549 cell line and significantly down-regulated the mRNA expression of RHEBL1 and up-regulated the mRNA expression of RNPC3, which was consistent with the bioinformatic predictions. These results confirm that RHEBL1 and RNPC3 may be potential targets for Polyphyllin VI and Protodioscin in the treatment of non-small cell lung cancer and may also be candidate diagnostic genes for determining the prognosis associated with non-small cell lung cancer.

The involvement of RhebL1 in sphingosylphosphorylcholine-induced events including (K8) phosphorylation, reorganization, migration and invasion was examined (Kim et al., 2017). In breast cancer cells MCF7, scholars found that overexpression of RhebL1 increased the expression of mesenchymal markers and decreased the expression of E-cadherin in MCF7 and that downregulation of this gene significantly reduced the migration and invasion of MCF7 cells (Kim and Lee, 2018). Several studies also found that high expression of exogenous RHEBL1 promoted the growth of malignant mesothelioma cells and probed that RHEB-mTORC1 signaling has a pro-carcinogenic effect (Bonneau and Parmar, 2012; Sato et al., 2021). In terms of RNPC3, little is known about the role of RNPC3 protein in human cancers, and some studies have shown that genes including RNPC3 can be used as diagnostic genes for lung adenocarcinoma (Li et al., 2020). In addition, RNPC3 rearrangements have been associated with the development of B-type acute lymphoblastic leukemia (Chen et al., 2020).

Several limitations remain in this study, although we investigated the correlation between patients with non-small cell lung cancer and immune infiltration, the immune infiltration results were not further validated. Secondly, Polyphyllin VI and Protodioscin promoted apoptosis and cell cycle arrest in A549 cells, which may be related to the expression profile of the characteristic genes RHEBL1 and RNPC3, however, the molecular mechanisms and roles involved need to be further investigated. Finally, the analysis of RHEBL1 and RNPC3 as potential targets and prognostic genes in this study was mainly based on the mRNA level, and no further in-depth analysis was done at the protein level to make the data more convincing. In conclusion, our findings strongly suggest that RHEBL1 and RNPC3 are potential targets for Polyphyllin VI and Protodioscin in the treatment of non-small cell lung cancer, and may also be candidate diagnostic genes for non-small cell lung cancer-related prognosis.

## Conclusion

In conclusion, we explored two potential key genes (RHEBL1 and RNPC3) for NSCLC by machine learning and bioinformatics approaches. We further validated by molecular docking and *in vitro* experiments that PPVI and Prot may act on RHEBL1 and RNPC3 to affect NSCLC. Additionally, this study provides new insights into the clinical treatment and molecular mechanisms of NSCLC, revealing potential therapeutic targets.

## Data Availability

The original contributions presented in the study are included in the article/Supplementary Material, further inquiries can be directed to the corresponding author/s.
