# Supplementary material for: Machine learning and bioinformatics-based insights into the potential targets of saponins in Paris polyphylla smith against non-small cell lung cancer
Source: Front Genet. 2022 Oct 28;13:1005896. doi: 10.3389/fgene.2022.1005896 (PMC9649596; doi:10.3389/fgene.2022.1005896)
Supplement: Supplementary file 1 [file DataSheet1.docx]

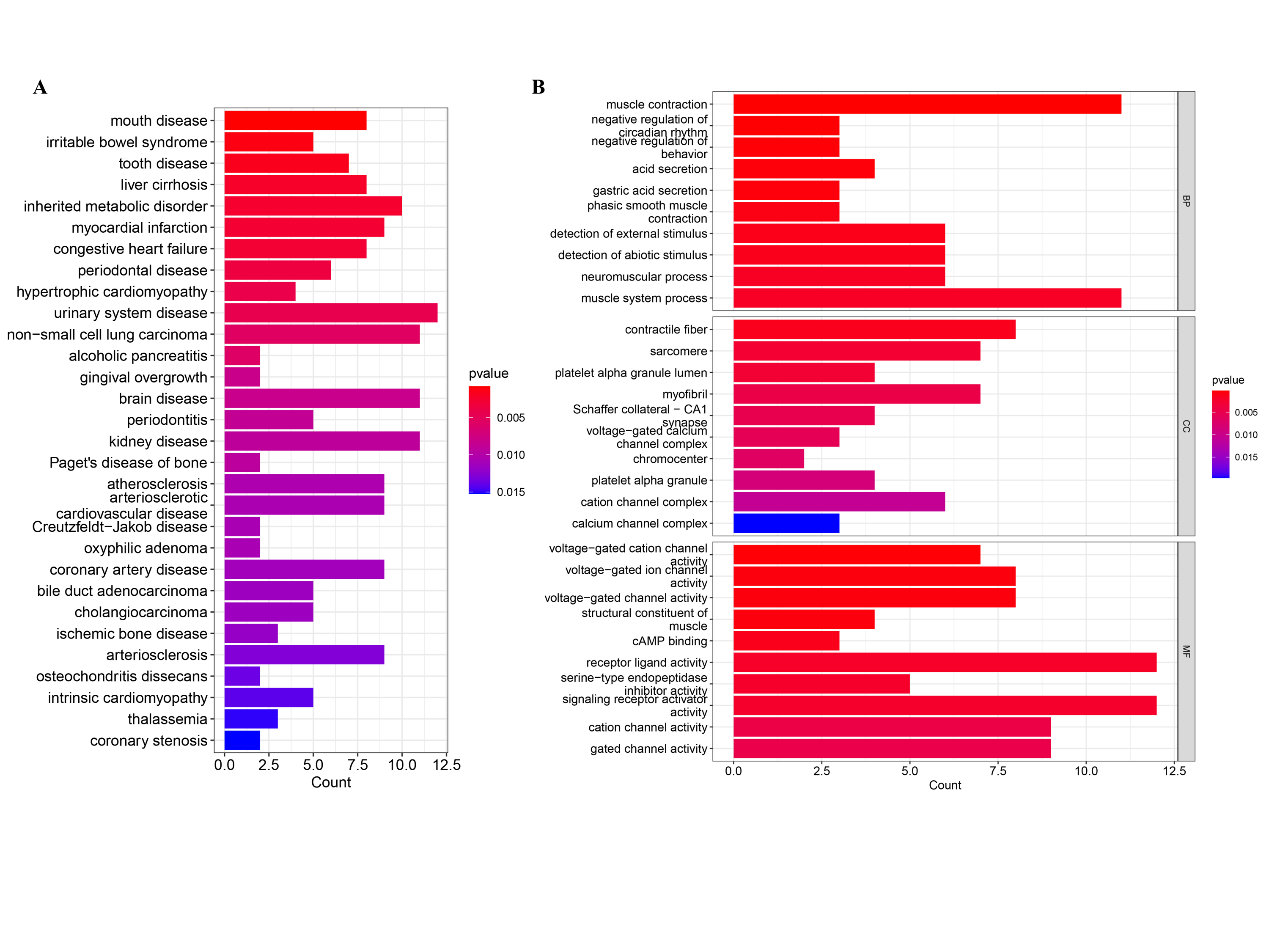


**Figure S1.** DO and GO enrichment analysis (A) DO analysis of the top 30 disease categories associated with differential genes; (B) GO analysis of the signaling pathways enriched in BP, MF and CC in NSCLC (CC: cellular component, MF: molecular function, BP: biological process)

**Figure S2.** Lasso regression analysis screens for differential genes associated with NSCLC

**Figure S3.** Results of SVM-RFE screening for non-small cell lung cancer-related signature genes

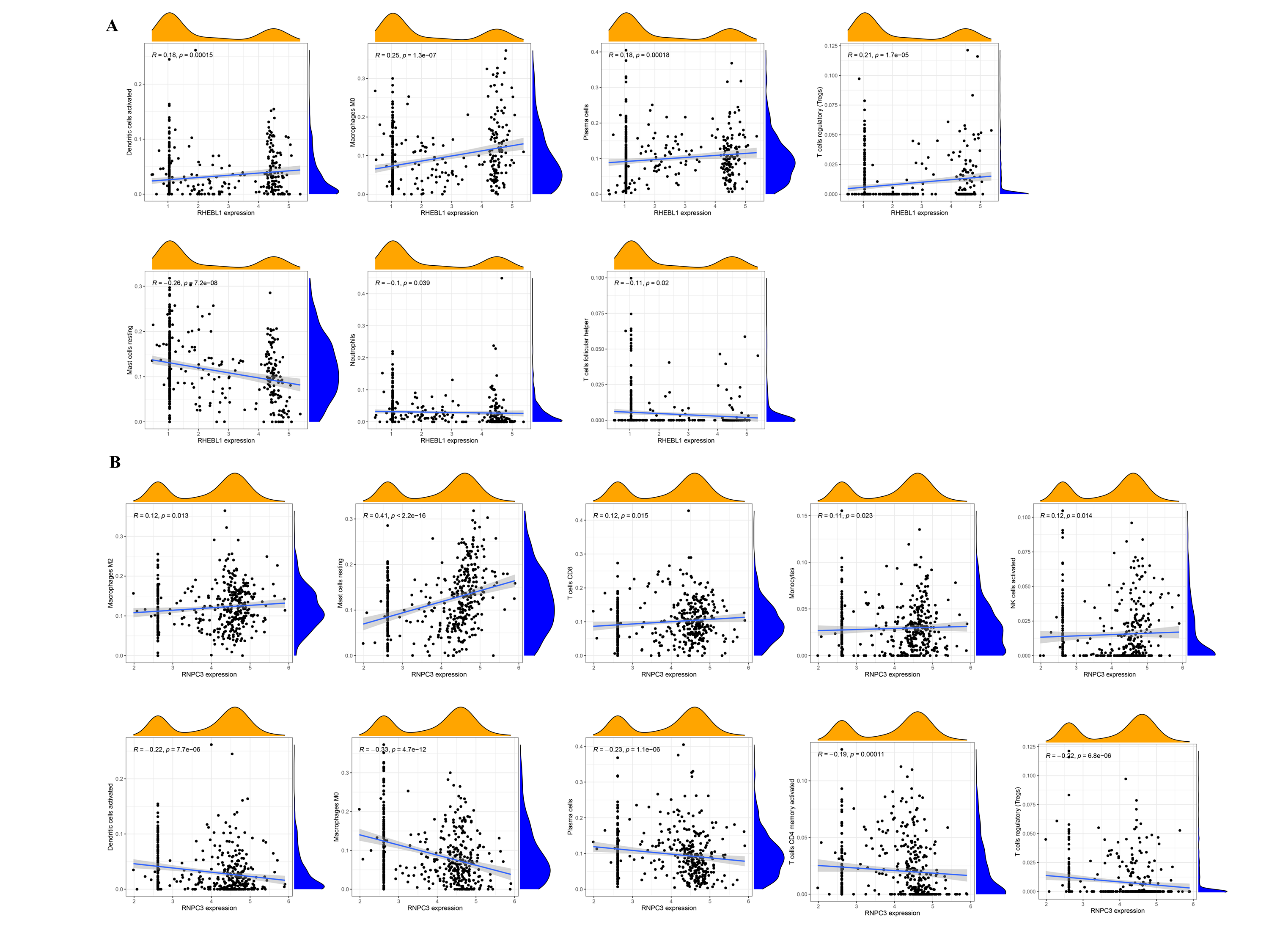


**Figure S4.** Correlation analysis of RNPC, RHEBL1 and immune cells (a) Correlation analysis of RHEBL1 and various types of immune cells (b) Correlation analysis of RNPC3 and various types of immune cells

**Table S1.** saponins in *Paris polyphylla* smith used for molecular docking

| **Serial Number** | **Name** | **Compound CID** | **Molecular Formula** | **Molecular Weight** |
| --- | --- | --- | --- | --- |
| **1** | **Polyphyllin H** | **101615586** | **C44H70O17** | **871.00** |
| **2** | **Polyphyllin VII** | **71307572** | **C51H84O22** | **1049.20** |
| **3** | **methyl protogracillin** | **44566783** | **C52H86O23** | **1079.20** |
| **4** | **Dioscin** | **119245** | **C45H72O16** | **869.00** |
| **5** | **Gracillin** | **159861** | **C45H72O17** | **885.00** |
| **6** | **Protodioscin** | **441891** | **C51H84O22** | **1049.20** |
| **7** | **Protogracillin** | **441892** | **C51H84O23** | **1065.20** |
| **8** | **Polyphyllin V** | **101377612** | **C39H62O12** | **722.90** |
| **9** | **Polyphyllin VI** | **10417550** | **C39H62O13** | **738.90** |
| **10** | **Pennogenin** | **12314056** | **C27H42O4** | **430.60** |
| **11** | **Polyphyllin I** | **11018329** | **C44H70O16** | **855.00** |
| **12** | **Polyphyllin II** | **46200821** | **C44H70O16** | **855.00** |
| **13** | **Parisaponin I** | **46877361** | **C50H82O22** | **1035.20** |
| **14** | **Prosapogenin A** | **11061578** | **C39H62O12** | **722.90** |
| **15** | **Pseudoproto-Pb** | **70688903** | **C57H92O25** | **1177.30** |
